# The genome sequence of the giant willow aphid, *Tuberolachnus salignus *(Gmelin, 1790)

**DOI:** 10.12688/wellcomeopenres.20646.1

**Published:** 2024-02-19

**Authors:** Liam M. Crowley, James McCulloch, Reuben James, University of Oxford and Wytham Woods Genome Acquisition Lab, Darwin Tree of Life Barcoding collective, Wellcome Sanger Institute Tree of Life Management, Samples and Laboratory team, Wellcome Sanger Institute Scientific Operations: Sequencing Operations, Wellcome Sanger Institute Tree of Life Core Informatics team, Tree of Life Core Informatics collective, Darwin Tree of Life Consortium

**Affiliations:** 1 Department of Biology, University of Oxford, Oxford, England, UK; 2 Hogenhout Lab, John Innes Centre Department of Crop Genetics, Norwich, England, UK

**Keywords:** Tuberolachnus salignus, giant willow aphid, genome sequence, chromosomal, Hemiptera

## Abstract

We present a genome assembly from an individual female *Tuberolachnus salignus* (the giant willow aphid; Arthropoda; Insecta; Hemiptera; Aphididae). The genome sequence is 456.8 megabases in span. Most of the assembly is scaffolded into 10 chromosomal pseudomolecules. The mitochondrial genome has also been assembled and is 22.43 kilobases in length.

## Species taxonomy

Eukaryota; Metazoa; Eumetazoa; Bilateria; Protostomia; Ecdysozoa; Panarthropoda; Arthropoda; Mandibulata; Pancrustacea; Hexapoda; Insecta; Dicondylia; Pterygota; Neoptera; Paraneoptera; Hemiptera; Sternorrhyncha; Aphidomorpha; Aphidoidea; Aphididae; Lachninae; *Tuberolachnu*s; *Tuberolachnus salignus* (Gmelin, 1790) (NCBI:txid96551).

## Background

The giant willow aphid, *Tuberolachnus salignus* (Gmelin, 1790), is one of the largest aphids (5 mm), and is easily identifiable due to its large size and the conical tubercle on the dorsum ( Blackman & Eastpop, 1994). Their bodies are black in colour with a mesh-like grey covering which gives them a uniformly spotty appearance. Their legs are red and black.

Giant willow aphids feed on willow species ( Blackman & Eastpop, 1994). In the UK, willow is used as a biomass crop, and the damage caused by the aphids reduces yield ( Aradottir *et al.*, 2012). Further, wild Bees feed on the honeydew they produce, which has been shown to reduce the success of bee activity, reducing pollination in important crops ( Sopow *et al.*, 2017). Therefore, gaining an understanding of the giant willow aphid is important to enable management strategies to protect important crops.

Giant willow aphids reproduce and feed on a single host and are presumed to only reproduce parthenogenically as no males have ever been found ( Blackman & Eastpop, 1994; Sopow *et al.*, 2017). Very few individuals have been recorded in the spring and it remains unclear where they go or if an alternative host is used during this period. Recently, colonies were found feeding on quince, *Cydonia oblonga* Mill., in May ( Salisbury, 2022). They are thought to have originated in Asia, but now have a worldwide distribution, predominantly found in Europe, including the UK and Ireland. More recently, they have been discovered in Australia and New Zealand ( Sopow *et al.*, 2017). Perhaps due to their asexual reproduction, there is little genetic diversity between different populations; a study on 27 populations in 5 countries, found only 16 genotypes ( Aradottir *et al.*, 2012).

The giant willow aphid’s large size makes it a useful model for aphid study. It is more amenable than smaller aphids to microinjections for the study of electrophysiology, the effects of RNAi molecules, and genetic manipulation. The genome of this aphid will enable more such studies.

We present a chromosomally complete genome sequence for *Tuberolachnus salignus* (Gmelin, 1790), based on one female specimen from Withymead Nature Reserve as part of the Darwin Tree of Life Project. This project is a collaborative effort to sequence all named eukaryotic species in the Atlantic Archipelago of Britain and Ireland.

## Genome sequence report

**Figure 1.**
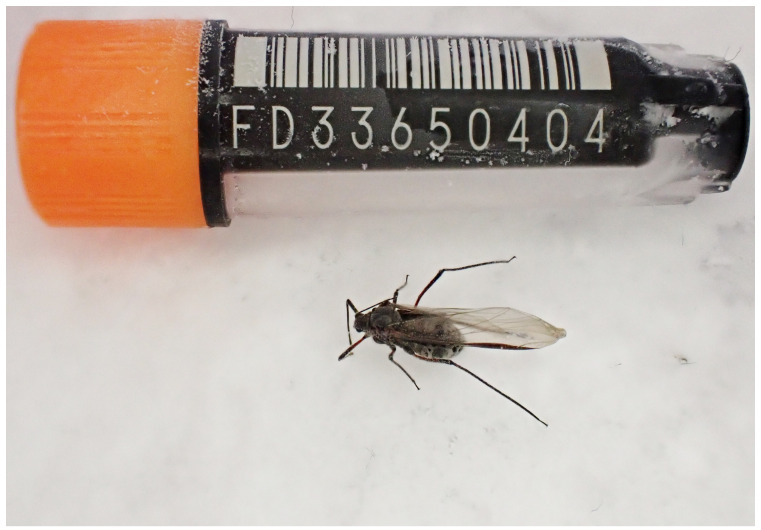
Photograph of the *Tuberolachnus salignus* (ihTubSali1) specimen used for genome sequencing.

The genome was sequenced from one female *Tuberolachnus salignus* ( Figure 1) collected from Withymead Nature Reserve, Oxfordshire, UK (51.54, –1.14). A total of 37-fold coverage in Pacific Biosciences single-molecule HiFi long reads was generated. Primary assembly contigs were scaffolded with chromosome conformation Hi-C data. Manual assembly curation corrected 84 missing joins or mis-joins and removed 3 haplotypic duplications, reducing the scaffold number by 39.02%, and increasing the scaffold N50 by 7.69%.

**Figure 2.**
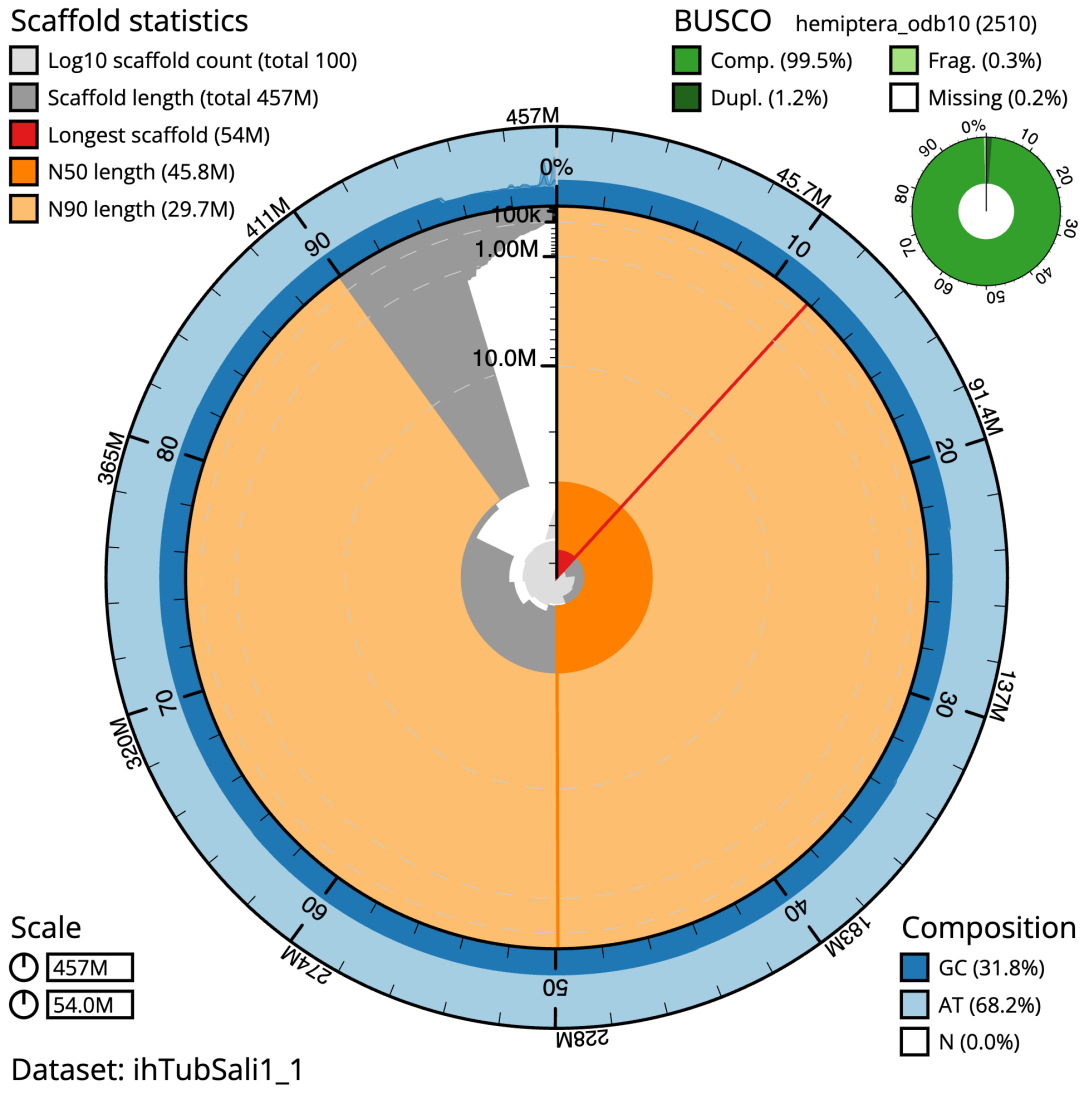
Genome assembly of *Tuberolachnus salignus*, ihTubSali1.1: metrics. The BlobToolKit Snailplot shows N50 metrics and BUSCO gene completeness. $BTK_SNAIL_LEG An interactive version of this figure is available at https://blobtoolkit.genomehubs.org/view/ihTubSali1_1/dataset/ihTubSali1_1/snail.

**Figure 3.**
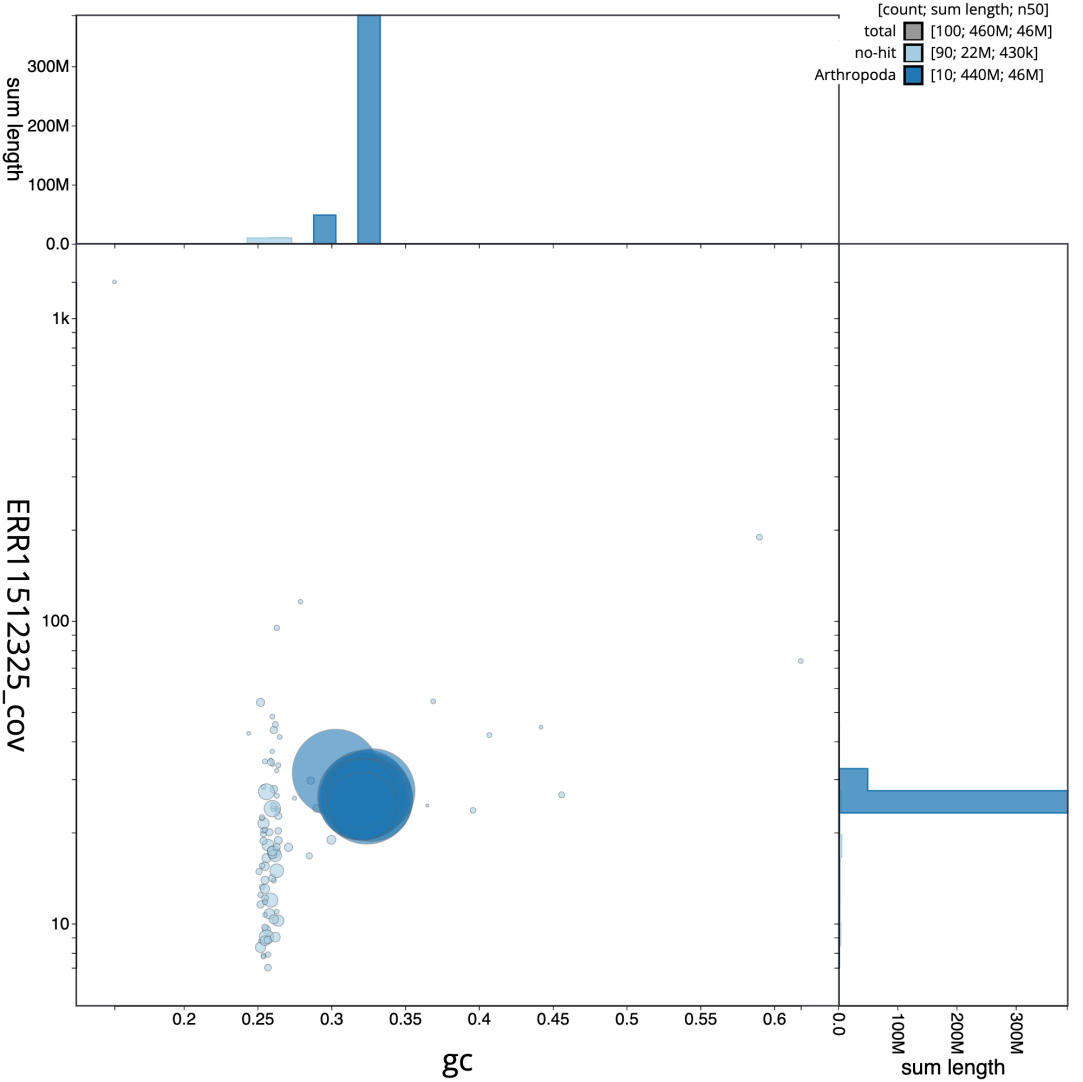
Genome assembly of *Tuberolachnus salignus*, ihTubSali1.1: BlobToolKit GC-coverage plot. Scaffolds are coloured by phylum. Circles are sized in proportion to scaffold length. Histograms show the distribution of scaffold length sum along each axis. An interactive version of this figure is available at https://blobtoolkit.genomehubs.org/view/ihTubSali1_1/dataset/ihTubSali1_1/blob.

**Figure 4.**
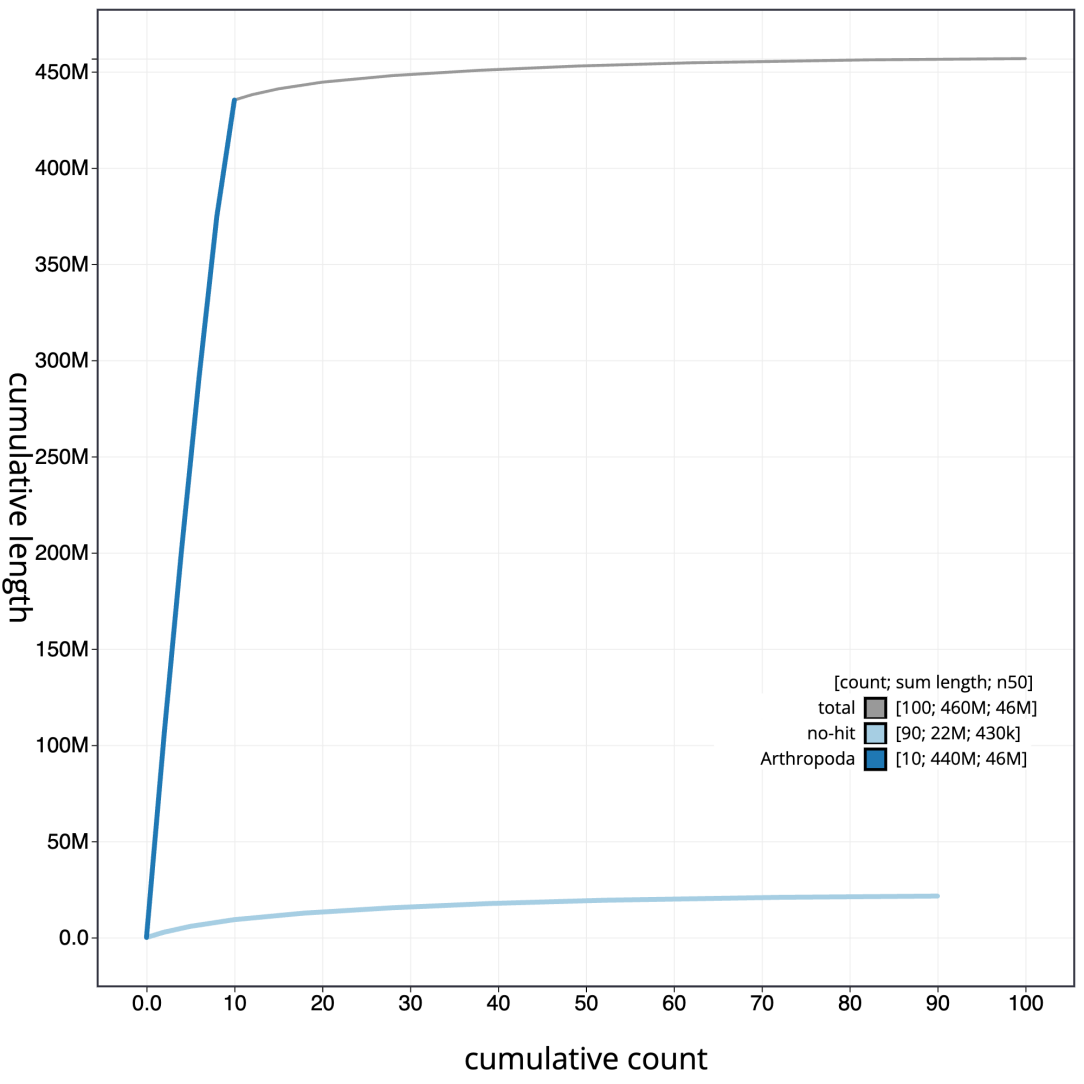
Genome assembly of *Tuberolachnus salignus*, ihTubSali1.1: BlobToolKit cumulative sequence plot. The grey line shows cumulative length for all scaffolds. Coloured lines show cumulative lengths of scaffolds assigned to each phylum using the buscogenes taxrule. An interactive version of this figure is available at https://blobtoolkit.genomehubs.org/view/ihTubSali1_1/dataset/ihTubSali1_1/cumulative.

**Figure 5.**
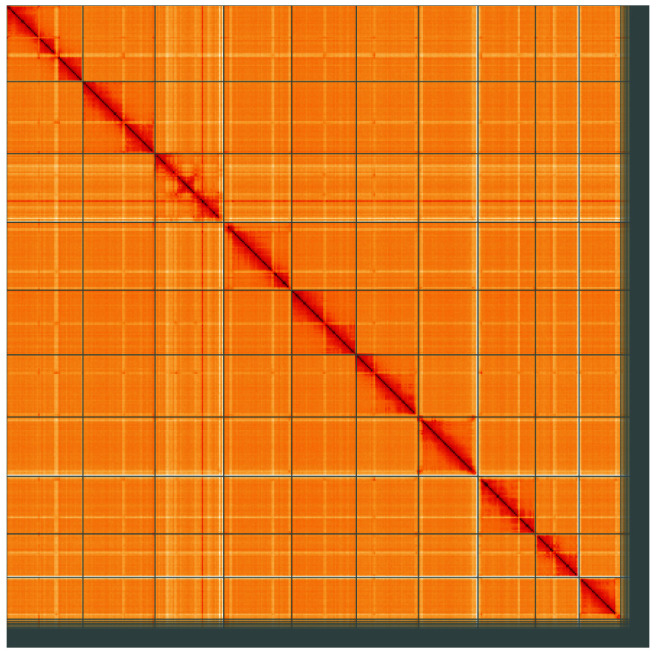
Genome assembly of *Tuberolachnus salignus*, ihTubSali1.1: Hi-C contact map of the ihTubSali1.1 assembly, visualised using HiGlass. Chromosomes are shown in order of size from left to right and top to bottom. An interactive version of this figure may be viewed at https://genome-note-higlass.tol.sanger.ac.uk/l/?d=GyIhnLv2RNi-c1J2QqK93A.

The final assembly has a total length of 456.8 Mb in 99 sequence scaffolds with a scaffold N50 of 45.8 Mb ( Table 1). The snailplot in Figure 2 provides a summary of the assembly statistics, while the distribution of assembly scaffolds on GC proportion and coverage is shown in Figure 3. The cumulative assembly plot in Figure 4 shows curves for subsets of scaffolds assigned to different phyla. Most (95.29%) of the assembly sequence was assigned to 10 chromosomal-level scaffolds, representing 10 autosomes. Chromosomes 3 and 7 align to the *Myzus persicae* X chromosome ( Mathers *et al.*, 2021), but no male data available to confirm X chromosome. Chromosome-scale scaffolds confirmed by the Hi-C data are named in order of size ( Figure 5; Table 2). While not fully phased, the assembly deposited is of one haplotype. Contigs corresponding to the second haplotype have also been deposited. The mitochondrial genome was also assembled and can be found as a contig within the multifasta file of the genome submission.

**Table 1.** Genome data for *Tuberolachnus salignus*, ihTubSali1.1.

Project accession data		
Assembly identifier	ihTubSali1.1	
Species	*Tuberolachnus salignus*	
Specimen	ihTubSali1	
NCBI taxonomy ID	96551	
BioProject	PRJEB62742	
BioSample ID	SAMEA112232857	
Isolate information	ihTubSali1, female: whole organism (DNA sequencing) ihTubSali2, female: whole organism (Hi-C scaffolding)	
Assembly metrics *		*Benchmark*
Consensus quality (QV)	59.4	*≥ 50*
*k*-mer completeness	100.0%	*≥ 95%*
BUSCO **	C:99.5%[S:98.3%,D:1.2%],F:0.3%, M:0.2%,n:2,510	*C ≥ 95%*
Percentage of assembly mapped to chromosomes	95.29%	*≥ 95%*
Sex chromosomes	None	*localised homologous pairs*
Organelles	Mitochondrial genome: 22.43 kb	*complete single alleles*
Raw data accessions		
PacificBiosciences SEQUEL II	ERR11512325	
Hi-C Illumina	ERR11526215	
Genome assembly		
Assembly accession	GCA_956483605.1	
*Accession of alternate haplotype*	GCA_956483625.1	
Span (Mb)	456.8	
Number of contigs	266	
Contig N50 length (Mb)	5.5	
Number of scaffolds	99	
Scaffold N50 length (Mb)	45.8	
Longest scaffold (Mb)	54.03	

* Assembly metric benchmarks are adapted from column VGP-2020 of “Table 1: Proposed standards and metrics for defining genome assembly quality” from ( Rhie *et al.*, 2021). ** BUSCO scores based on the hemiptera_odb10 BUSCO set using version 5.3.2. C = complete [S = single copy, D = duplicated], F = fragmented, M = missing, n = number of orthologues in comparison. A full set of BUSCO scores is available at https://blobtoolkit.genomehubs.org/view/ihTubSali1_1/dataset/ihTubSali1_1/busco.

**Table 2.** Chromosomal pseudomolecules in the genome assembly of *Tuberolachnus salignus*, ihTubSali1.

INSDC accession	Chromosome	Length (Mb)	GC%
OY101429.1	1	54.03	32.5
OY101430.1	2	51.1	32.5
OY101431.1	3	48.61	30.5
OY101432.1	4	48.22	32.0
OY101433.1	5	45.84	33.0
OY101434.1	6	44.03	32.5
OY101435.1	7	42.02	32.0
OY101436.1	8	40.81	32.5
OY101437.1	9	30.88	32.0
OY101438.1	10	29.69	32.0
OY101439.1	MT	0.02	15.5

The estimated Quality Value (QV) of the final assembly is 59.4 with *k*-mer completeness of 100.0%, and the assembly has a BUSCO v5.3.2 completeness of 99.5% (single = 98.3%, duplicated = 1.2%), using the hemiptera_odb10 reference set ( *n* = 2,510).

Metadata for specimens, barcode results, spectra estimates, sequencing runs, contaminants and pre-curation assembly statistics are given at https://links.tol.sanger.ac.uk/species/96551.

## Methods

### Sample acquisition and nucleic acid extraction

Two female *Tuberolachnus salignus* was collected from Withymead Nature Reserve, Oxfordshire, UK (latitude 51.54, longitude –1.14) on 2022-08-12 using a sweep net. The specimen was collected by Liam Crowley and James McCulloch (University of Oxford) and identified by Liam Crowley and preserved on dry ice. One specimen was used for DNA sequencing (specimen ID Ox002686, ToLID ihTubSali1) and the other (specimen ID Ox002687, ToLID ihTubSali2) was used for Hi-C sequencing.

Protocols developed by the Wellcome Sanger Institute (WSI) Tree of Life core laboratory have been deposited on protocols.io ( Denton *et al.*, 2023b). The workflow for high molecular weight (HMW) DNA extraction at the WSI includes a sequence of core procedures: sample preparation; sample homogenisation, DNA extraction, fragmentation, and clean-up. In sample preparation, the ihTubSali1 sample was weighed and dissected on dry ice ( Jay *et al.*, 2023). Tissue from the whole organism was homogenised using a PowerMasher II tissue disruptor ( Denton *et al.*, 2023a). HMW DNA was extracted in the WSI Scientific Operations core using the Automated MagAttract v2 protocol ( Oatley *et al.*, 2023). HMW DNA was sheared into an average fragment size of 12–20 kb in a Megaruptor 3 system with speed setting 31 ( Bates *et al.*, 2023). Sheared DNA was purified by solid-phase reversible immobilisation ( Strickland *et al.*, 2023): in brief, the method employs a 1.8X ratio of AMPure PB beads to sample to eliminate shorter fragments and concentrate the DNA. The concentration of the sheared and purified DNA was assessed using a Nanodrop spectrophotometer and Qubit Fluorometer and Qubit dsDNA High Sensitivity Assay kit. Fragment size distribution was evaluated by running the sample on the FemtoPulse system.

### Sequencing

Pacific Biosciences HiFi circular consensus DNA sequencing libraries were constructed according to the manufacturers’ instructions. DNA sequencing was performed by the Scientific Operations core at the WSI on a Pacific Biosciences SEQUEL II instrument. Hi-C data were also generated from tissue of the whole organism of ihTubSali2 using the Arima2 kit and sequenced on the Illumina NovaSeq 6000 instrument.

### Genome assembly, curation and evaluation

Assembly was carried out with Hifiasm ( Cheng *et al.*, 2021) and haplotypic duplication was identified and removed with purge_dups ( Guan *et al.*, 2020). The assembly was then scaffolded with Hi-C data ( Rao *et al.*, 2014) using YaHS ( Zhou *et al.*, 2023). The assembly was checked for contamination and corrected as described previously ( Howe *et al.*, 2021). Manual curation was performed using HiGlass ( Kerpedjiev *et al.*, 2018) and Pretext ( Harry, 2022). The mitochondrial genome was assembled using MitoHiFi ( Uliano-Silva *et al.*, 2023), which runs MitoFinder ( Allio *et al.*, 2020) or MITOS ( Bernt *et al.*, 2013) and uses these annotations to select the final mitochondrial contig and to ensure the general quality of the sequence.

A Hi-C map for the final assembly was produced using bwa-mem2 ( Vasimuddin *et al.*, 2019) in the Cooler file format ( Abdennur & Mirny, 2020). To assess the assembly metrics, the *k*-mer completeness and QV consensus quality values were calculated in Merqury ( Rhie *et al.*, 2020). This work was done using Nextflow ( Di Tommaso *et al.*, 2017) DSL2 pipelines “sanger-tol/readmapping” ( Surana *et al.*, 2023a) and “sanger-tol/genomenote” ( Surana *et al.*, 2023b). The genome was analysed within the BlobToolKit environment ( Challis *et al.*, 2020) and BUSCO scores ( Manni *et al.*, 2021; Simão *et al.*, 2015) were calculated.

Table 3 contains a list of relevant software tool versions and sources.

**Table 3.** Software tools: versions and sources.

Software tool	Version	Source
BlobToolKit	4.2.1	https://github.com/blobtoolkit/blobtoolkit
BUSCO	5.3.2	https://gitlab.com/ezlab/busco
Hifiasm	0.16.1-r375	https://github.com/chhylp123/hifiasm
HiGlass	1.11.6	https://github.com/higlass/higlass
Merqury	MerquryFK	https://github.com/thegenemyers/MERQURY.FK
MitoHiFi	3	https://github.com/marcelauliano/MitoHiFi
PretextView	0.2	https://github.com/wtsi-hpag/PretextView
purge_dups	1.2.5	https://github.com/dfguan/purge_dups
sanger-tol/genomenote	v1.0	https://github.com/sanger-tol/genomenote
sanger-tol/readmapping	1.1.0	https://github.com/sanger-tol/readmapping/tree/1.1.0
YaHS	1.2a.2	https://github.com/c-zhou/yahs

### Wellcome Sanger Institute – Legal and Governance

The materials that have contributed to this genome note have been supplied by a Darwin Tree of Life Partner. The submission of materials by a Darwin Tree of Life Partner is subject to the **‘Darwin Tree of Life Project Sampling Code of Practice’**, which can be found in full on the Darwin Tree of Life website here. By agreeing with and signing up to the Sampling Code of Practice, the Darwin Tree of Life Partner agrees they will meet the legal and ethical requirements and standards set out within this document in respect of all samples acquired for, and supplied to, the Darwin Tree of Life Project. 

Further, the Wellcome Sanger Institute employs a process whereby due diligence is carried out proportionate to the nature of the materials themselves, and the circumstances under which they have been/are to be collected and provided for use. The purpose of this is to address and mitigate any potential legal and/or ethical implications of receipt and use of the materials as part of the research project, and to ensure that in doing so we align with best practice wherever possible. The overarching areas of consideration are:

• Ethical review of provenance and sourcing of the material

• Legality of collection, transfer and use (national and international) 

Each transfer of samples is further undertaken according to a Research Collaboration Agreement or Material Transfer Agreement entered into by the Darwin Tree of Life Partner, Genome Research Limited (operating as the Wellcome Sanger Institute), and in some circumstances other Darwin Tree of Life collaborators.

## Data Availability

European Nucleotide Archive: *Tuberolachnus salignus* (giant willow aphid). Accession number PRJEB62742; https://identifiers.org/ena.embl/PRJEB62742 ( Wellcome Sanger Institute, 2023). The genome sequence is released openly for reuse. The *Tuberolachnus salignus* genome sequencing initiative is part of the Darwin Tree of Life (DToL) project. All raw sequence data and the assembly have been deposited in INSDC databases. The genome will be annotated using available RNA-Seq data and presented through the Ensembl pipeline at the European Bioinformatics Institute. Raw data and assembly accession identifiers are reported in Table 1.

## Author information

Members of the University of Oxford and Wytham Woods Genome Acquisition Lab are listed here: https://doi.org/10.5281/zenodo.7125292.

Members of the Darwin Tree of Life Barcoding collective are listed here: https://doi.org/10.5281/zenodo.4893703.

Members of the Wellcome Sanger Institute Tree of Life Management, Samples and Laboratory team are listed here: https://doi.org/10.5281/zenodo.10066175.

Members of Wellcome Sanger Institute Scientific Operations: Sequencing Operations are listed here: https://doi.org/10.5281/zenodo.10043364.

Members of the Wellcome Sanger Institute Tree of Life Core Informatics team are listed here: https://doi.org/10.5281/zenodo.10066637.

Members of the Tree of Life Core Informatics collective are listed here: https://doi.org/10.5281/zenodo.5013541.

Members of the Darwin Tree of Life Consortium are listed here: https://doi.org/10.5281/zenodo.4783558.
