# Supplementary material for: The genome sequence of the giant willow aphid, Tuberolachnus salignus (Gmelin, 1790)
Source: Wellcome Open Res. 2024 Feb 19;9:59. [Version 1] doi: 10.12688/wellcomeopenres.20646.1 (PMC11303955; doi:10.12688/wellcomeopenres.20646.1)
Supplement: Peer review history

# Reviewer report: Reviewer response for version 1

**DOI:** 10.21956/wellcomeopenres.22850.r90144

**Published:** 2024-08-06

**Authors:** Kay Lucek

**Affiliations:** 1 University of Neuchâtel, Neuchâtel, Switzerland

The authors present the chromosome level genome assembly of a female specimen of the giant willow aphid *Tuberolachnus salignus*. The assembly consists of 10 chromosomes, where the identity of the X chromosome is unclear as no males have so far been found for this species. The assembly is highly complete as revealed by the high BUSCO score but not fully phased. Sequencing and genome assembly follow the current state of the art and use established methods.

 Overall, the presented assembly will be of great value to study genome evolution in aphids and will enable applied projects. However, as with other genome notes of the Darwin Tree of Life project, the genome is not yet annotated. Instead the data availability statement highlights that “The genome will be annotated using available RNA-Seq data and presented through the Ensembl pipeline at the European Bioinformatics Institute.”, without giving a reference to the RNAseq data.

 The species description section is very informative.

Are sufficient details of methods and materials provided to allow replication by others?

Yes

Is the rationale for creating the dataset(s) clearly described?

Yes

Are the datasets clearly presented in a useable and accessible format?

Yes

Are the protocols appropriate and is the work technically sound?

Yes

Reviewer Expertise:

Speciation genomics

I confirm that I have read this submission and believe that I have an appropriate level of expertise to confirm that it is of an acceptable scientific standard.

# Reviewer report: Reviewer response for version 1

**DOI:** 10.21956/wellcomeopenres.22850.r86460

**Published:** 2024-07-15

**Authors:** Zachary Cohen

**Affiliations:** 1 Insect Control and Cotton Disease Research Unit, Southern Plains Agricultural Research Center,Agricultural Research Service, United States Department of Agriculture, College Station, TX, USA

This genome report is satisfactory but there are some grammatical typos that need to be corrected. 

 Background:

 # Third sentence, bees shouldn't be capitalized: Further, wild Bees feed on the honeydew they produce 

 # next line, fourth sentence add genetic to specify the understanding of this aphid species. Therefore, gaining a GENETIC understanding

 parthenogenically misspelled 

 # Third paragraph, sentence three, specify where the colonies were found on quince.

 Methods:

 # first line, it should be females and were collected: Two female *s* *Tuberolachnus salignus* were collected...etc.

 # next line, plural specimen and agreement: The specimen *s* *were*

Are sufficient details of methods and materials provided to allow replication by others?

Yes

Is the rationale for creating the dataset(s) clearly described?

Yes

Are the datasets clearly presented in a useable and accessible format?

Yes

Are the protocols appropriate and is the work technically sound?

Yes

Reviewer Expertise:

insect genetics, genomics, evolution, agricultural adaptation.

I confirm that I have read this submission and believe that I have an appropriate level of expertise to confirm that it is of an acceptable scientific standard.
